# Colopathy associated with pentosan polysulfate use

**DOI:** 10.3389/fphar.2025.1494467

**Published:** 2025-07-28

**Authors:** Emily H. Jung, Wei Zheng, Ryan J. Weiss, Namita E. Mathew, Benjamin I. Meyer, Azhar Nizam, Heba Iskandar, Nieraj Jain

**Affiliations:** ^1^ Department of Ophthalmology, Duke University School of Medicine, Durham, NC, United States; ^2^ Department of Pathology and Laboratory Medicine, Emory University School of Medicine, Atlanta, GA, United States; ^3^ Complex Carbohydrate Research Center, University of Georgia, Athens, GA, United States; ^4^ Department of Biochemistry and Molecular Biology, University of Georgia, Athens, GA, United States; ^5^ Department of Ophthalmology & Vision Science, The University of Arizona, Tucson, AZ, United States; ^6^ Bascom Palmer Eye Institute, University of Miami Leonard M. Miller School of Medicine, Miami, FL, United States; ^7^ Department of Biostatistics and Bioinformatics, Biostatistics Collaboration Core, Emory University, Atlanta, GA, United States; ^8^ Department of Medicine, Division of Digestive Diseases, Emory University School of Medicine, Atlanta, GA, United States; ^9^ Department of Ophthalmology, Emory University School of Medicine, Atlanta, GA, United States

**Keywords:** pentosan polysulfate, elmiron, interstitial cystitis, inflammatory bowel disease, irritable bowel syndrome, toxicity, dextran sodium sulfate, colopathy

## Abstract

**Introduction:**

We describe a novel colopathy associated with pentosan polysulfate (PPS) use and assess the strength of the drug-disease association in a two-part investigation.

**Methods:**

1. Cohort Study: We studied individuals with a history of long-term PPS use. Case histories concerning gastrointestinal disease were obtained with review of endoscopy records and histopathology specimens. Findings were summarized with descriptive statistics. 2. Cross-Sectional Study: We evaluated patients with interstitial cystitis at a single clinical center. We obtained data on drug exposure and medical histories and measured the strength of association between PPS use and diagnosis of inflammatory bowel disease (IBD) using multivariate logistic regression.

**Results:**

1. Cohort Study: Among 13 participants, the median PPS exposure was 2.04 kg (range 0.99–2.54 kg). Eleven participants (84.6%) developed IBD symptomatology after initiating PPS therapy, and 9 (69.2%) were diagnosed with IBD. Two others (18%) were diagnosed with irritable bowel syndrome. Of the 10 participants with endoscopic and histopathologic data, six had abnormal colonic mucosa on endoscopy, and all 10 had histologic abnormalities. Clinical and histologic improvement was noted after PPS cessation, though two (18%) required colectomy for colitis-associated dysplasia. 2. Cross-Sectional Study: Among 219 subjects with interstitial cystitis, PPS use was a statistically significant predictor of an IBD diagnosis, with an adjusted odds ratio of 3.3 (95% confidence interval, 1.2–8.8, p = 0.02).

**Discussion:**

Our study identifies a strong association between PPS use and clinical diagnosis of IBD. Histopathologic findings suggest a novel drug-associated colopathy, with some subjects necessitating colectomy for dysplasia. Further investigation into the causality of this association is warranted.

## Introduction

Interstitial cystitis (IC), also known as bladder pain syndrome, is a chronic condition characterized by suprapubic and pelvic pain as well as lower urinary tract symptoms. It affects more than one million U.S. adults, predominantly women (Clemens et al., 2022). Although the pathophysiology of IC remains unclear, abnormalities in the bladder uroepithelium may expose the bladder wall to irritants within the urine (McLennan, 2014). Under the 2022 American Urological Association guidelines, there are four recommended oral medications for the treatment of IC (Clemens et al., 2022). Of these, pentosan polysulfate (PPS) [ELMIRON, Janssen Pharmaceuticals, Titusville, NJ] is the only oral drug approved by the U.S. Food and Drug Administration (FDA) for management of IC (Giusto et al., 2018).

Pentosan polysulfate is a semi-synthetic sulfated polysaccharide that is structurally related to the cellular glycosaminoglycan heparan sulfate. It is postulated that PPS may integrate into and support the inner lining of the bladder urothelium (Janssen Pharmaceuticals, 2021). Pharmacokinetic studies estimate that approximately 0.14% of an oral dose of PPS (300 or 450 mg) reaches the bladder as intact drug, while 58%–84% passes through the colon and is excreted in feces as intact drug (Janssen Pharmaceuticals, 2021). PPS received FDA approval for IC management in 1996 and has been associated with a number of adverse events, with diarrhea (3.9%), alopecia (3.9%), and nausea (3.7%) being the most frequently reported in the original FDA medical review documents (U.S. Food and Drug Administration, 1996). Five of the 33 serious adverse drug reactions were gastrointestinal (GI) in nature and attributed to an osmotic load effect induced by undigested or unabsorbed PPS. Notably, the FDA medical review noted that the disease IC itself is associated with an increased incidence of inflammatory bowel disease (IBD) (U.S. Food and Drug Administration, 1996; Koziol, 1994). Indeed, studies show that approximately 2% of patients with classic IC also have IBD, compared to a 0.07% prevalence in the general population (Peeker et al., 2003; Alagiri et al., 1997).

**FIGURE 1:**
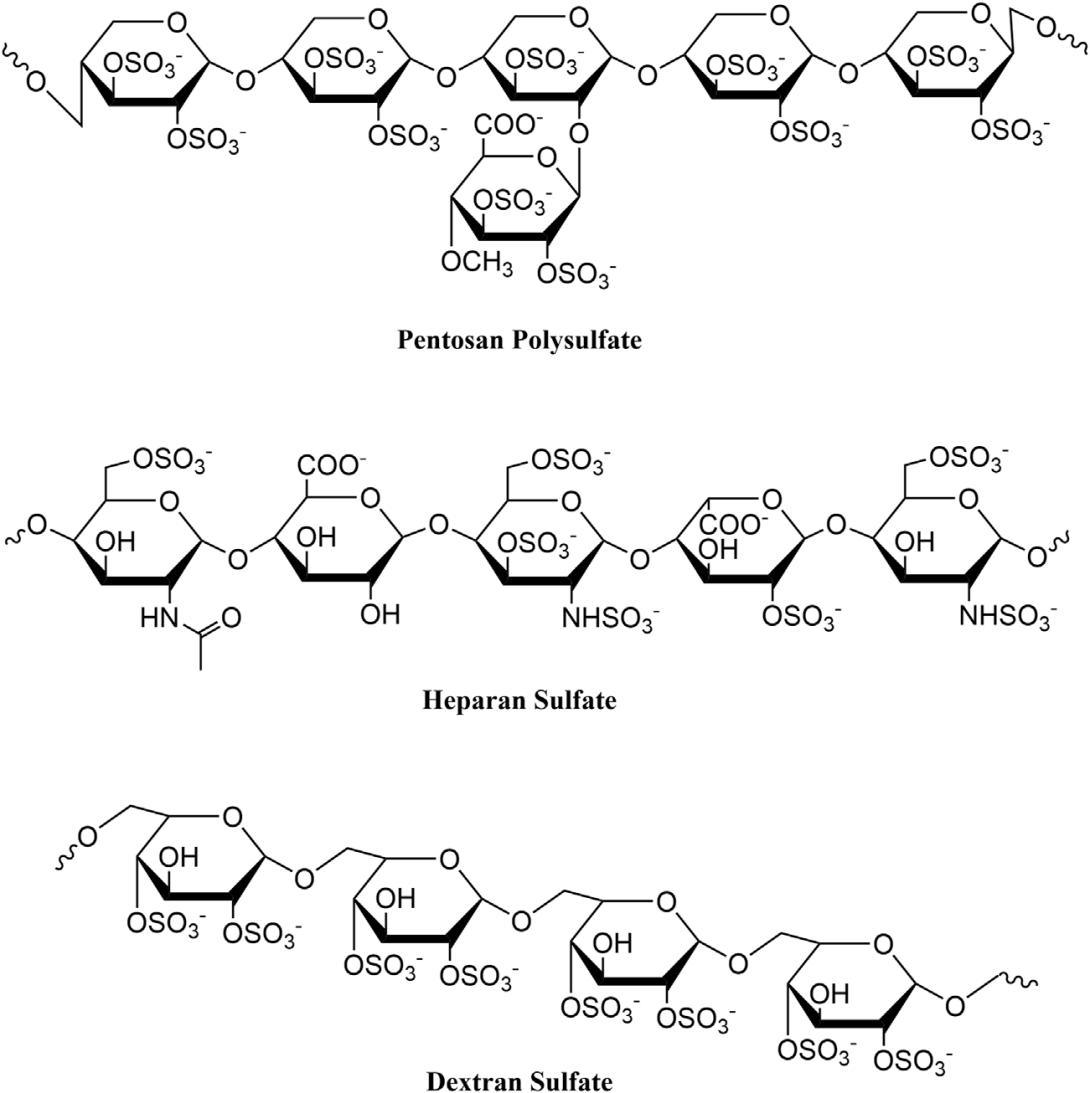
Chemical structures of pentosan polysulfate, heparan sulfate, and dextran sulfate.

In 2018, our group described a novel vision-threatening retinal disease associated with long-term PPS use, which subsequently led to a drug labeling change (Pearce et al., 2018, Hanif et al., 2019, Hall et al., 2025). During our ongoing investigations of PPS associated retinal disease, we identified a high prevalence of IBD, with many patients reporting “confusing” or “atypical” diagnoses from prior GI evaluations (Jung et al., 2023). Intriguingly, dextran sodium sulfate (DSS), a structurally related compound, is routinely used to artificially induce acute and chronic colitis in animal models (Figure 1) for the study of IBD (Chassaing et al., 2014; Kiesler et al., 2015). DSS, like PPS, is a semi-synthetic sulfated polysaccharide, a class of drugs known to have a range of potentially beneficial and detrimental biological activities (e.g., anticoagulant, antioxidant, immunomodulatory) (Wang et al., 2018), due in part to high negative charge density and tendency to interface with cell surfaces (Arlov et al., 2021).

We hypothesize that the GI diseases observed in our PPS-exposed patients may represent a drug toxicity. Of note, a recent retrospective series identified a high rate of colonic dysplasia in patients with IBD who had used PPS (Anderson et al., 2022). Herein, we report our explorations into a novel association between PPS use and incident colopathy with a cohort study of participants with long-term PPS exposure. Additionally, to expand on observations from the cohort study, we perform a cross-sectional investigation of individuals with IC to further evaluate the association between PPS exposure and incident colopathy (Supplementary Figure S1).

## Methods

The study was conducted at Emory University (Atlanta, GA) and received approval from the Emory University Institutional Review Board. The study followed the tenets set forth by the Declaration of Helsinki.

### Part I: Cohort study

Fourteen participants with PPS associated retinal disease enrolled in a four-year prospective study of their ocular and systemic health, prior to our concerns regarding an association between PPS and colopathy. The study protocol was subsequently amended to permit more detailed evaluations of systemic health. All participants had discontinued PPS use prior to the baseline visit. Study enrollment occurred between 2019 and 2020. All participants provided written informed consent and Health Insurance Portability and Accountability Act authorization.

Although the study protocol did not include formal GI evaluation, all available medical records pertaining to GI health were obtained. This included medical records pertaining to GI symptoms and diagnoses, endoscopy reports, and histopathology reports. Participants were queried for additional medical history and to verify content from the medical records. Biopsy specimens were retrieved for further evaluation by an expert gastrointestinal pathologist (WZ). Each case was reviewed by a gastroenterologist with expertise in inflammatory bowel disease (HI). Demographic and clinical characteristics were summarized using descriptive statistics. Note that because participants exhibited a range of colonic pathologies that did not always fit typical IBD diagnostic criteria, we use the broader ‘colopathy’ term where appropriate to describe spectrum of colonic disease associated with PPS use in this study.

### Part II: Cross-sectional study

To further explore the strength of association between PPS and GI disease, a cross-sectional study was conducted among individuals with a history of IC seen at our facility. The requirement for informed consent for this portion of the study was waived. Local billing records, electronic medical records, and pharmacy databases were queried for patients with a diagnosis of IC seen at the Emory Eye Center between May 2014 and October 2018. For each patient with IC, all available Emory Healthcare medical records were reviewed to identify those with a diagnosis of IBD and/or irritable bowel syndrome (IBS). Records were also evaluated for exposure to PPS and other IC therapies as well as medications associated with the development or exacerbation of IBD.

Odds ratios (OR) were computed to quantify the association between each drug exposure and two separate outcomes based on clinical diagnoses in the medical record: IBD, and a composite outcome of IBD and/or IBS (IBD + IBS). A composite IBD + IBS outcome was assessed because many participants received unclear and sometimes varying clinical diagnoses by their gastroenterologist, often due to atypical presentations that did not fit a classic IBD presentation. Crude (unadjusted) associations were assessed as follows: two-sample t-tests were used to compare the average age for participants with and without the outcomes, and either chi-square tests or, where appropriate, Fisher’s exact tests were used for associations between categorical covariates and the outcomes. Multivariable logistic regression was performed for both outcomes, controlling for known predictors of IBD (age, gender, smoking status, and nonsteroidal anti-inflammatory drug (NSAID) use) while assessing the impact of PPS and other IC medication exposures. Adjusted ORs were estimated and p-values for type III tests determined. Significance level was set at p < 0.05 (confidence level 95%). Statistical analysis was performed using SAS version 9.4 (SAS Institute, Cary, NC).

## Results

### Cohort study

Fourteen individuals with PPS associated retinal disease were enrolled in a prospective study of their ocular and systemic health (Table 1). One individual withdrew following the baseline visit and was excluded from all analyses. All 13 included participants had a longstanding diagnosis of IC previously managed with PPS and all experienced gastrointestinal symptoms while on PPS. Twelve (92.3%) were female and twelve (92.3%) were White. Median (interquartile range [IQR]) age at study enrollment was 62 years (48–65). Median cumulative PPS exposure was 2.04 kg (0.99–2.54) over a median duration of 14 years (9–18).

**TABLE 1:** Demographics and PPS exposure history by study subject (cohort study).

Subject ID	Age/Sex/Race	Body Mass index (kg/m^2^)	Year of IC diagnosis	Duration of PPS intake (years)	Mean PPS daily dose (mg)	Cumulative PPS Exposure (kg)	Smoking history	NSAID use
1	38/F/W	38.1	2003	14	400	2.04	Never	
2	65/F/W	26.2	2011	7.4	300	0.84	Never	
3	68/F/W	27.8	1994	19.9	300	2.18	Never	
4	63/F/W	29.0	1998	17	400	2.48	Never	Y (Daily use for >10 years)
5	44/F/W	26.2	2014	3	400	0.44	Never	
6	48/F/W	35.4	2003	14	400	2.04	Never	
7	62/F/W	20.4	1978	12	300	1.31	Never	
8	54/F/W	21.1	1995	18.3	400	2.68	Never	
9	37/F/AA	22.2	1999	9	300	0.99	Never	
10	72/F/W	19.3	1995	21.8	400	3.19	Never	
11	76/M/W	26.7	2000	19.8	400	2.85	7-pack-year; quit at age 21	
12	51/F/W	25.2	2007	6.4	300	0.70	Never	
13	64/F/W	25.1	2002	17.4	400	2.54	Never	

Eleven (84.6%) participants experienced the onset of GI symptoms following the initiation of PPS therapy (Table 2). Median time between PPS therapy initiation and symptom onset was 4.9 years (3.5–6.2). Initial symptoms included diarrhea (n = 7), abdominal pain (n = 2), stool with mucus and blood (n = 2), tenesmus (i.e., sensation of needing to defecate) (n = 1), fecal urgency (n = 1), and fecal incontinence (n = 1). For the two participants with symptom onset prior to PPS use, one (subject 1) was diagnosed with lactose intolerance at age nine and one (subject 6) had received a prior diagnosis of IBS.

**TABLE 2:** Gastrointestinal history and disease course by study subject (cohort study).

		GI symptoms									GI diagnoses^a^			
Subject ID	Duration between PPS initiation and initial GI symptoms (years)	Abdominal pain	Constipation	Diarrhea	Incontinence	Mucus in stool	Hematochezia	Tenesmus	Urgency	Duration between PPS initiation and earliest abnormal histopathologic findings (years)	IBD	IBS	GI surgical history	IBD medications
1	−1.3					x^b^				-			Abdominal lipoma tumor removal	
2	4.3		x^b^	x						4.9	UC, MC		Colectomy (colitis-associated dysplasia)	Mesalamine (discontinued)
3	7.8	x					x	x	x	8.2	CD		Cholecystectomy	Balsalazide
4	5.3			x						17.3	CD	x	Cholecystectomy, adhesiolysis	Budesonide
5	0.8		x^c^	x						3.0	Indeterminate		Appendectomy, sigmoid colectomy (fixed loop of sigmoid colon in the pelvis following hysterectomy)	
6	−4.4	x^b^	x^b^					x^b^		-		x		
7	0.8			x						1.5	UC			
8	7.0	x		x			x			7.0	UC			Mesalamine (discontinued)
9	3.0									-		x	Appendectomy	
10	4.0			x						21.5		x	Cholecystectomy	
11	4.9			x						15.7	UC		Colectomy (colitis-associated dysplasia)	Mesalamine (discontinued)
12	5.0			x						5.9	UC			Mesalamine
13	9.9				x					11.4	UC			Mesalamine

^a^ = diagnoses were extracted from gastroenterologist reports.

^b^ = symptoms occurred prior to starting PPS.

^c^ = constipation was attributed to a fixed loop of sigmoid colon in the pelvis.

IBD = Crohn’s disease (CD), microscopic colitis (MC), ulcerative colitis (UC), and non-specific colitis.

Abbreviations: ID, identification; PPS, pentosan polysulfate; GI, gastrointestinal; IBD, inflammatory bowel disease; IBS, irritable bowel syndrome; UC, ulcerative colitis; MC, microscopic colitis; CD, Crohn’s disease.

All 11 participants with symptom onset after starting PPS underwent at least one colonoscopy while on the drug, and all 11 received a clinical diagnosis of IBD and/or IBS by their gastroenterologist. Nine participants received a diagnosis of IBD, including five with ulcerative colitis (UC), two with Crohn’s disease, one with microscopic colitis, and one with indeterminate colitis. Three of the 11 participants were diagnosed with IBS, one of whom also had an IBD diagnosis.

**FIGURE 2:**
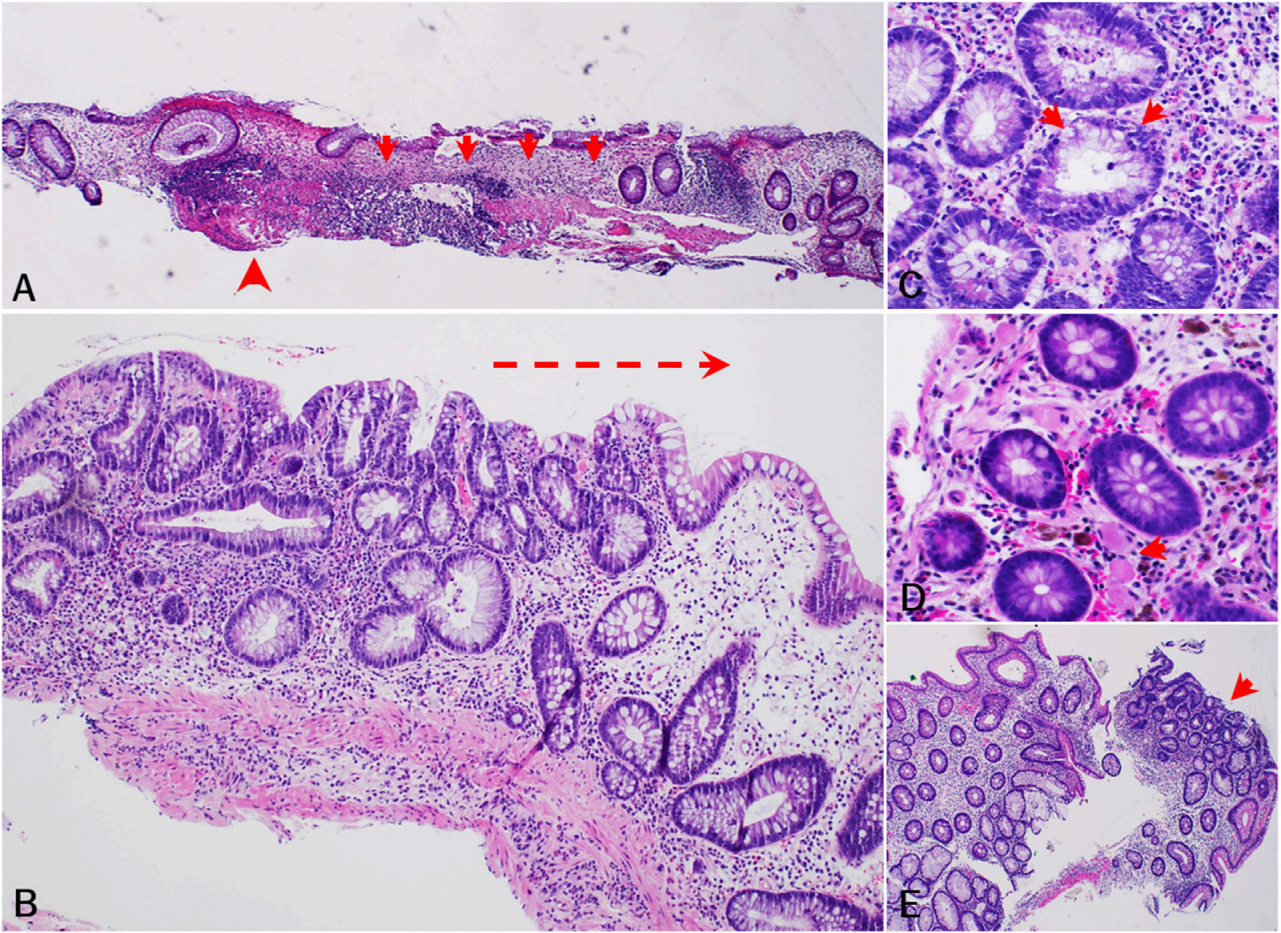
Histopathologic findings of PPS-related mucosal injury of colon (cohort study). **(A)** [subject 5, 2015] Chronic mucosal injury characterized by apparent crypt dropout (arrow) and architectural disarray, patchy lamina propria fibrosis, and muscularis mucosa thickening (arrowhead). **(B)** [subject 11, 2016] Injured colonic mucosa showing crypt architectural distortion and increased lamina propria lymphoplasmacytic inflammation with increased eosinophils and scattered neutrophils. Note lymphoplasmacytic inflammation progressing from left to right (arrow). **(C)** [subject 4, 2018] Increased crypt epithelial apoptotic bodies. **(D)** [subject 8, 2013] Fibrin-appearing eosinophilic amorphous deposition in lamina propria capillaries (arrow) and/or some stromal cells. Note melanosis coli also present. **(E)** [subject 11, 2015] Low grade dysplasia (arrow) in non-targeted colonic biopsy. Note background colonic mucosa exhibiting crypt architecture abnormalities and regenerative changes.

Endoscopy reports, pathology reports, and histology slides were available for 10 of these 11 participants (Supplementary Table S1). On endoscopy, three (27.3%) of the 11 had both mucosal ulcerations and adenomas, one (9.1%) had mucosal ulcerations only, two had adenomas only (18.2%), and four (36.4%) had normal-appearing colonic mucosa. Histology showed abnormal mucosal changes in all 10 participants whose reports and slides we reviewed. Median time from PPS initiation to the earliest abnormal histopathologic finding was 7.6 years (5.2–14.6). Seven (63.6%) participants exhibited chronic mucosal injury and five (45.5%) had histologic findings of focal minimal to mild active colitis. Three (27.3%) had only chronic mucosal injury with no significant lamina propria inflammation and two (18.2%) had only reactive changes. Specific findings included crypt architectural distortion (n = 8), patchy lamina propria fibrosis and thickening of the muscularis mucosae (n = 4), patchy lamina propria lymphoplasmacytic inflammation with increased eosinophils (n = 5), increased crypt epithelial apoptotic bodies (n = 4), fibrin-appearing eosinophilic amorphous deposition in lamina propria capillaries and/or stromal cells (n = 2), foamy cytoplasm (n = 2), superficial granulomas (n = 1), and low-grade dysplasia (n = 1) (Figure 2; Supplementary Table S1).

All 11 participants reported their symptoms have since improved. Seven of the nine with IBD received medical therapy. Symptoms improved with mesalamine (n = 5), balsalazide (n = 1), and budesonide (n = 1). Three participants, who had all been using mesalamine, have since discontinued the medication and have not started alternative medications. One participant (subject 11) stopped using mesalamine following a colectomy, one (subject 2) stopped 7 months after discontinuing PPS, and one (subject 8) stopped while still on PPS therapy. All 11 participants have discontinued PPS therapy.

Four participants (subjects 2, 3, 8, 12) had serial colonic biopsy specimens, including while on PPS and after stopping PPS therapy (Supplementary Table S1). Histopathologic signs of active colitis were observed for three of these participants (subjects 3, 8, 12) on PPS, none of which were evident in the biopsy specimens obtained when they were off therapy. All three managed their GI symptoms with mesalamine. Two (subjects 3, 12) of these subjects had rectosigmoid ulcerations on endoscopy, both of whose colonic lesions resolved following cessation of PPS use. These two participants (subjects 3, 12) also had histopathologic evidence of lamina propria lymphoplasmacytic inflammation while on PPS, which was no longer present when they were off therapy. The participant without histopathologic signs of active colitis (subject 2) while on PPS did have findings of chronic mucosal injury, which were no longer present on repeat evaluation when they were off PPS.

Two participants underwent a colectomy for colitis-associated dysplasia (characteristic precancerous epithelial changes) (Table 2). One participant (subject 11) underwent a colectomy for dysplasia 1 year after being diagnosed with UC, which was 15 years after starting PPS. This participant was on PPS at the time of the colectomy and had an approximate 2.42 kg cumulative exposure at that time. Another participant (subject 2) with a 0.84 kg cumulative PPS exposure had a partial colectomy for dysplasia in the context of microscopic colitis. This participant was initially diagnosed with UC 4.9 years into treatment with PPS but had been off PPS for 3.2 years prior to the colectomy. A third participant (subject 3) with a 2.18 kg cumulative PPS exposure is being monitored for a dysplastic lesion that may ultimately require partial colectomy.

Family history of IBD was present in one participant and colorectal cancer in three. Exposure to other IC treatments included hydroxyzine (n = 4), amitriptyline (n = 4), gabapentin (n = 3), cyclobenzaprine (n = 2), methenamine (n = 2), and phenazopyridine (n = 1), none of which have been implicated in drug-induced colitis. Some of the 11 participants reported chronic use of medications that are associated with colitis, including statins (n = 7), mesalamine (n = 5), and NSAIDs (n = 1) (Hamdeh et al., 2021).

### Cross-sectional study

During the study period, 219 patients with IC were seen at the Emory Eye Center. Mean age was 60.8 (standard deviation, 15.1) years and 195 (89.0%) subjects were female. PPS exposure was documented in 80 (36.5%) subjects and was a statistically significant predictor of an IBD diagnosis [adjusted OR = 3.3 (95% confidence interval, 1.2–8.8, p = 0.02)]. Similarly, PPS exposure was associated with the composite IBD + IBS outcome [adjusted OR = 3.3 (95% confidence interval, 1.5–7.3, p = 0.002)] (Table 3; Supplementary Table S2). No other medication exposure was associated with an increased risk of IBD.

**TABLE 3:** Study subject demographics and exposures to potential risk factors according to presence or absence of IBD, and IBD or IBS (cross-sectional study).

Covariate	IBD			IBD or IBS		
	Yes (n = 19) Mean ± SD or n (%)	No (n = 200) Mean ± SD or n (%)	Crude^a^ (C) and Adjusted^b^ (A) Odds ratios (95% CI), P-value	Yes (n = 34) Mean ± SD or n (%)	No (n = 185) Mean ± SD or n (%)	Crude^a^ (C) and Adjusted^b^ (A) Odds ratios (95% CI), P-value
Age^c^ (years)	59.2 ± 11.8	60.9 ± 15.4	C: 1.0 (0.8, 1.1), 0.60 A: 1.0 (1.0, 1.0), 0.83	57.3 ± 14.0	61.4 ± 15.3	C: 0.9 (0.8, 1.0), 0.10 A: 1.0 (0.8, 1.1), 0.51
Sex						
Female (n = 195)	18 (9%)	177 (91%)	C: 2.3 (0.3, 101.6), 0.70 A: 1.4 (0.2, 12.1), 0.74	32 (16%)	163 (84%)	C: 2.2 (0.5, 19.8), 0.39 A: 1.4 (0.3, 6.6), 0.69
Male (n = 24)	1 (4%)	23 (96%)		2 (8%)	22 (92%)	
Smoking^d^						
Never (n = 161)	17 (11%)	144 (89%)		29 (18%)	132 (82%)	
Current (n = 4)	0 (0%)	4 (100%)	C: 0.3 (0.1, 1.4), 0.10 A: 0.3 (0.1, 1.5), 0.14	0 (0%)	4 (100%)	C: 0.4 (0.2, 1.2), 0.09 A: 0.5 (0.2, 1.3), 0.15
Former (n = 54)	2 (4%)	52 (96%)		5 (9%)	49 (91%)	
PPS						
Yes (n = 80)	12 (15%)	68 (85%)	**C: 3.3 (1.3, 8.8), 0.01^e^ A: 3.3 (1.2, 8.8), 0.02**	21 (26%)	59 (74%)	**C: 3.4 (1.6, 7.4), <0.001 A: 3.3 (1.5, 7.3), 0.002**
No (n = 139)	7 (5%)	132 (95%)		13 (9%)	126 (91%)	
NSAIDs						
Yes (n = 165)	11 (7%)	154 (93%)	C: 0.4 (0.1, 1.3), 0.09 A: 0.4 (0.2, 1.2), 0.11	20 (12%)	145 (88%)	**C: 0.4 (0.2, 0.8), 0.02 A: 0.4 (0.2, 0.9), 0.03**
No (n = 54)	8 (15%)	46 (85%)		14 (26%)	40 (74%)	

^a^ For each of the ‘IBD’ and ‘IBD, or IBS’ outcomes, bivariate associations between the outcome and a covariate were assessed by estimating the crude odds ratio, computing a 95% confidence interval for the odds ratio and determining the p-value for the relevant test of association. Chi-square tests or Fisher’s Exact tests were used, as appropriate; where Fisher’s Exact test was used, exact confidence intervals were computed.

^b^ The association between PPS, and each of the ‘IBD’ and ‘IBD, or IBS’ outcomes was assessed using a binary logistic regression including PPS, age, sex, smoking status and NSAIDS, as predictors. Adjusted odds ratios were estimated from the regressions, 95% confidence intervals for the odds ratios computed and p-values for type III, tests of association determined.

^c^ The odds ratios for age compare the odds of the outcome based on a 5-year difference in age.

^d^ The odds ratios for smoking compare the odds of the outcome for current and former smokers with the odds for those who never smoked.

^e^ Statistically significant results are denoted in bold.

## Discussion

Our study demonstrates a novel association between exposure to PPS, a drug that has been widely used to manage IC for decades, and a possible drug-induced colopathy causing gastrointestinal symptoms. Among the 13 participants with high cumulative PPS exposure in our cohort study, 9 had received a clinical diagnosis of IBD by their gastroenterologist after initiating PPS therapy, and two underwent colectomy for dysplasia. Our cross-sectional study of 219 subjects with IC corroborates this association, demonstrating that PPS was the only significant predictor of an IBD diagnosis among all IC therapies evaluated, with an adjusted OR of 3.3.

Similar to our findings with PPS-associated retinal disease, this PPS-associated colopathy appears to manifest after long-term use of the drug, although further study is needed to establish a dose-response relationship. Symptoms ranging from diarrhea to pain and bleeding appeared to be moderate in severity but generally manageable with therapies such as mesalamine. Participants’ symptoms, need for treatment, and endoscopic and histopathologic findings all appeared to subside after PPS cessation. However, some patients were left with permanent sequelae after undergoing colectomy for colitis-associated precancerous lesions.

Histopathologically, seven of the ten participants with available histology slides exhibited chronic mucosal injury and five participants had findings of focal minimal to mild activity. Histopathological features of this PPS-associated colopathy included: 1) chronic mucosal injury characterized by crypt dropout and architectural disarray, and patchy lamina propria fibrosis and thickening of the muscularis mucosae, 2) patchy increased lamina propria lymphoplasmacytic inflammation with increased eosinophils, 3) increased crypt epithelial apoptotic bodies, 4) fibrin-appearing eosinophilic amorphous deposition in lamina propria capillaries and/or some stromal cells, and 5) foamy change of crypt epithelial cytoplasm and nonspecific superficial granulomas. These features are often observed in drug-induced colonic injury or colopathy (Püspök et al., 2000; Price, 2003). In some biopsies, low-grade dysplasia was also observed in non-adenoma cases (non-targeted colonic biopsies), with the background colonic mucosa exhibiting only mild crypt architecture irregularity and regenerative changes. However, classic diagnostic features of IBD, such as basal crypt branching and diffuse basal plasmacytosis with lifting of the base of the crypts from the muscularis mucosae, were not evident in any biopsies.

Although the pathobiology of a potential PPS-induced colopathy is unclear, it is worth noting that PPS is similar in chemical structure to dextran sodium sulfate (Figure 1), which is widely used in a chemically-induced model of colitis in mice (Chassaing et al., 2014). PPS contains repeating sulfated xylan residues (2 sulfate groups per xylose residue), with a molecular weight range of 4,000–7,000 Daltons. Similarly, DSS is a highly sulfated polysaccharide (∼2.3 sulfates per glucosyl residue), with a molecular weight range of 5,000–14,000 Daltons. Due to the high degree of sulfation, both compounds have a high negative charge that may account for their biologic activities. Notably, differences in degree of sulfation and sulfation patterning, monosaccharide composition, three-dimensional structure, and chain length may alter the pharmacological effect of each drug. Further, it is unclear whether PPS itself or a metabolite may be associated with both the retinal and gastrointestinal diseases.

Regarding the DSS colitis model, mice that received DSS for only 7 days developed an active chronic colitis histologically characterized by areas of both activity and inactivity, crypt loss and distortion, epithelial proliferation, and possibly even dysplasia (Cooper et al., 1993). The earliest histologic features that were observed in the DSS colitis model, such as crypt loss without accompanying inflammation, are similar to what was seen in our study. Furthermore, studies in rats have demonstrated that long term exposure to DSS and other sulfated polysaccharides have led to intestinal tumors (Ishioka et al., 1987).

Although this is the first study to provide a detailed exploration of the association between PPS use and new-onset colopathy, prior studies have suggested an increased incidence of lower GI symptoms among PPS users. Indeed, current FDA labeling of the drug includes GI-related findings from an unblinded trial of 2499 IC subjects treated with PPS for varying durations (Janssen Pharmaceuticals, 2021). “Colitis” is mentioned as one of 38 adverse events, at a frequency of ≤1%. The label cautions that patients with “gastrointestinal ulcerations, polyps, or diverticula” should be “carefully evaluated prior to starting ELMIRON” (Janssen Pharmaceuticals, 2021). In the FDA medical review for PPS, it was noted that future phase IV studies would further explore the question of whether the GI side effects seen among the study subjects were due to the drug or to underlying diseases (U.S. Food and Drug Administration, 1996). A phase IV study (NCT00086684) was initiated in September 2003 and 369 subjects were assigned to one of three intervention arms for 24 weeks (PPS 100 mg once a day, PPS 100 mg three times a day, and placebo). Citing the lack of safety concerns raised on interim analysis, the phase IV study was terminated in June 2011.

Other studies have also reported on adverse GI effects among PPS users. In a phase I study investigating PPS for advanced cancers, 20 of 21 subjects experienced grade 1 or 2 gastrointestinal bleeding (Marshall et al., 1997). Proctitis was the second most common toxicity, with nonspecific signs of inflammation on histology. Of note, in this study of subjects with multiple medical comorbidities, the use of concomitant medications, their potentially toxic effects, and potential drug interaction effects were not fully investigated. Another clinical study found that 4% of subjects receiving PPS 300 mg daily for 32 weeks experienced rectal hemorrhages (Nickel et al., 2005). The Anderson et al. retrospective study demonstrated high rates of colonic dysplasia among IBD patients who took PPS (Anderson et al., 2022). Notably, a majority of these cases had an IBD diagnosis before PPS initiation, and this study did not specifically evaluate the association between PPS use and new IBD diagnoses. Finally, another recent study identified a dose-response relationship between PPS exposure and a history of IBD (Santina et al., 2025).

Importantly, our findings do not establish a causal relationship between PPS and colopathy. An alternative explanation is that the underlying diagnosis of IC or another IC therapy is responsible for the GI disease. Indeed, multiple studies have demonstrated a link between IC and IBD (Peeker et al., 2003; Alagiri et al., 1997). However, the strong association demonstrated here with two distinct studies, suggestion of temporality, and plausibility shown through studies of DSS are concerning. Further, prior studies of IC yield a 2% IBD prevalence rate that is much lower than the 69.2% rate in our cohort study (Peeker et al., 2003; Alagiri et al., 1997). Finally, our cross-sectional study did not show evidence of confounding by any of the other IC therapies; PPS was the only drug with a statistically significant association with an IBD diagnosis.

Our cohort study was limited by its relatively small size and use of retrospective data. Clinical diagnoses were extracted from gastroenterology records. Given that not all cases diagnosed as ‘IBD’ fit a classic IBD presentation, we prefer the more generic ‘colopathy’ term to describe spectrum of colonic disease associated with PPS use in this study. Additionally, most colonoscopy procedures were performed prior to consideration of a possible medication side effect, and biopsies of the background colonic mucosa were not consistently available. Thus, we were unable to directly compare endoscopic and histologic features from time points preceding PPS use, during PPS therapy, and following cessation of the drug in every subject. Regarding the cross-sectional study, some relevant data regarding potential confounders, such as family history of IBD, were not available in our dataset. Despite these limitations, the consistent findings from these two separate investigations do provide a springboard for future study.

In conclusion, our study is suggestive of a novel association between PPS exposure and new onset colopathy. These findings have important public health implications, given that PPS has been widely prescribed for decades. Although this may be a reversible condition, there is a significant burden of disease that often prompts invasive diagnostic procedures and treatments, and development of precancerous lesions requiring colectomy in some participants suggests that more deleterious outcomes are possible. Further study is warranted to investigate a potential causal relationship, evaluate the clinical impact of this condition, and explore the pathobiology of the disease.

## Data Availability

The data analyzed in this study is subject to the following licenses/restrictions: The data underlying this article cannot be shared publicly due to the privacy of individuals who participated in the study. Requests to access these datasets should be directed to nieraj.jain@emory.edu.
