# Supplementary material for: Colopathy associated with pentosan polysulfate use
Source: Front Pharmacol. 2025 Jul 28;16:1494467. doi: 10.3389/fphar.2025.1494467 (PMC12336275; doi:10.3389/fphar.2025.1494467)
Supplement: Supplementary file 1 [file Table1.docx]

**Supplemental Table 1.** Endoscopy and histology findings by study subject (cohort study).

|  | | | | **Endoscopy Findings** | | | | **Histology Findings** | | | |
| --- | --- | --- | --- | --- | --- | --- | --- | --- | --- | --- | --- |
| **Subject ID** | **Date of PPS initiation** | **Date of PPS cessation** | **Date and indication for colonoscopy** | **Polyps** | **Mucosal erythema/erosions** | **Aphthous ulcerations** | **Macroscopic colitis-appearing mucosa** | **Crypt distortion/dropout/branching** | **Eosinophils (E) and neutrophils (N)** | **Thickening of the muscularis mucosae** | **Other findings and general histopathologic comments** |
| 1 | 5/2004 | 5/2018 | - | - | - | - | - | - | - | - | - |
| 2 | 9/2011 | 2/2019 | 10/2005  Constipation | 1 | 0 | 0 | 0 | n/a (only polyps biopsied) | n/a | n/a |  |
|  |  |  | **8/2016**  **Diarrhea** | **1** | **0** | **0** | **0** | **1** | **0** | **0** | **Mild chronic mucosal injury with no significant inflammation.** |
|  |  |  | 9/2019  UC/MC history, screening/asymptomatic | 1 | 0 | 0 | 0 | 0 | 0 | 0 | Benign colonic mucosa demonstrating no significant histopathologic change. |
|  |  |  | *10/2020  Screening/asymptomatic | 1 | 0 | 0 | 0 | n/a (only polyps biopsied) | n/a | n/a |  |
|  |  |  | *12/2021  Screening/asymptomatic | 1 | 0 | 0 | 0 | n/a (only polyps biopsied) | n/a | n/a |  |
| 3 | 8/1998 | 7/2018 | **10/2006**  **Hematochezia** | **0** | **1** | **1** | **1** | **1** | **N** | **1** | **Acute and chronic focally crypt-destructive colitis (lymphoplasmacytic inflammation). Mildly increased crypt epithelial apoptotic bodies. Definitive granulomata are not identified.** |
|  |  |  | **1/2007**  **CD history** | **0** | **1** | **1** | **1** | **1** | **E** | **0** | **Epithelium is slightly hypermucinous. Inflammatory cell population within the lamina propria is not increased, encroaching, or shifted toward acuity. Minimal crypt architectural changes and focal mild active chronic inflammation.** |
|  |  |  | **5/2010**  **CD history** | **0** | **n/a** | **n/a** | **n/a** | **1** | **N** | **0** | **Hypermucinous crypts. One fragment with fairly prominent lymphoid nodule with a germinal center. Mild active chronic inflammation and architectural disorder, nonspecific.** |
|  |  |  | 8/2021  CD history | 1 | 0 | 0 | 0 | 0 | 0 | 0 | Colonic mucosa with melanosis coli; otherwise no significant histopathology. No evidence of active, chronic, or microscopic colitis.  No granulomas identified. |
|  |  |  | 9/2021  In 8/2021, performed piecemeal removal of adenoma | 1 | 0 | 0 | 0 | 1 | 0 | 0 | Colonic mucosal villous change suggestive of chronic mucosal injury. |
|  |  |  | 6/2022  CD history | 1 | 0 | 0 | 0 | 1 | 0 | 0 | No granulomas are identified, no increase in inflammation. Colonic mucosa with patchy mild architectural irregularity/regenerative features, suggesting healing from prior injury. |
| 4 | 9/2001 | 9/2018 | *12/2018  Diarrhea | 0 | 0 | 0 | 0 | 1 | E | 1 | Chronic colonic mucosal injury characterized by crypt dropout, architectural disarray. Patchy lamina propria fibrosis and thickening of the muscularis mucosae. Mildly increased crypt epithelial apoptotic bodies. Patchy increased lamina propria eosinophils. Small nonspecific superficial granulomas in the proximal colon. |
| 5 | 4/2012 | 4/2015 | **6/2012**  **Specimen obtained from sigmoidectomy** | **0** | **0** | **0** | **0** | **0** | **0** | **0** | **Reactive colonic mucosa with occasional small lymphoid aggregates. Ganglion cells present in the intermyenteric Auerbach’s plexus.** |
|  |  |  | ***4/2015**  **Diarrhea** | **0** | **0** | **0** | **0** | **1** | **E** | **1** | **Chronic colonic mucosal injury characterized by crypt dropout, architectural disarray. Patchy lamina propria fibrosis and thickening of the muscularis mucosae. Mildly increased crypt epithelial apoptotic bodies. Reactive ileal mucosa.** |
| 6 | 6/2004 | 6/2018 | - | - | - | - | - | - | - | - | - |
| 7 | 3/2006 | 3/2018 | **9/2007**  **Diarrhea** | **0** | **1** | **1** | **1** | **1** | **E,N** | **0** | **Histologic features in all three biopsies are similar with the sigmoid colon more pronounced. Edema is present within the lamina propria of all three specimens as well as some reactive architectural glandular changes. Sigmoid colon biopsy shows a moderate increase in inflammatory cells in the lamina propria consisting of lymphocytes, plasma cells, eosinophils, and neutrophils.** |
| 8 | 1/2001 | 5/2019 | **2008**  **Diarrhea and hematochezia** | **n/a** | **n/a** | **n/a** | **0** | **n/a** | **n/a** | **n/a** | **No procedure/pathology reports available. GI clinic note states no evidence of active disease was noted on colonoscopy.** |
|  |  |  | ***10/2013**  **UC history** | **0** | **0** | **0** | **0** | **0** | **0** | **0** | **Fibrin-appearing eosinophilic amorphous deposition in lamina propria capillaries and/or some stromal cells. Melanosis coli. Focally increased reactive histiocytes in the lamina propria.** |
|  |  |  | ***2/2019**  **Screening/asymptomatic** | **0** | **0** | **0** | **0** | **0** | **0** | **0** | **Multiple mucosal lymphoid aggregates and occasional foamy cytoplasm of stromal and epithelial cells. No inclusion bodies/globules.** |
|  |  |  | *9/2019  Screening/asymptomatic | 0 | 0 | 0 | 0 | 0 | 0 | 0 | No lymphoid aggregates, no foamy cytoplasm, and no inclusion bodies/globules. |
| 9 | 1/1999 | 1/2008 | - | - | - | - | - | - | - | - | - |
| 10 | 1/1996 | 11/2017 | ***7/2017**  **Diarrhea** | **0** | **0** | **0** | **0** | **0** | **E,N** | **0** | **Focal lamina propria lymphoplasmacytic inflammation with an increase in eosinophils, lymphoid aggregates, and focal red blood cell extravasation.** |
| 11 | 2/2000 | 10/2019 | **2012**  **Diarrhea** | **n/a** | **n/a** | **n/a** | **0** | **n/a** | **n/a** | **n/a** | **No procedure/pathology reports available. GI clinic note states no evidence of active disease was noted on colonoscopy.** |
|  |  |  | ***10/2015**  **Diarrhea** | **1** | **n/a** | **n/a** | **n/a** | **1** | **N** | **0** | **Low grade dysplasia arising in a background of chronic mucosal injury. Foamy cytoplasm noted** |
|  |  |  | ***2/2016**  **UC history** | **1** | **n/a** | **n/a** | **n/a** | **1** | **E,N** | **0** | **Crypt architectural distortion. Patchy increased lamina propria lymphoplasmacytic inflammation with an increase in eosinophils and scattered neutrophils. Mildly increased crypt epithelial apoptotic bodies. Indefinite for dysplasia in the inflamed areas.** |
| 12 | 1/2008 | 6/2014 | ***11/2013**  **Diarrhea** | **0** | **1** | **1** | **1** | **1** | **E,N** | **0** | **Colonic mucosa with dense inflammation consisting of lymphocytes, histiocytes, neutrophils, and scattered eosinophils.** |
|  |  |  | *12/2014  UC history | 0 | 0 | 0 | 0 | 0 | 0 | 0 | Chronic colonic mucosal injury. Fibrin-appearing eosinophilic amorphous deposition in the lamina propria capillaries. |
|  |  |  | 9/2018  UC history | 1 | 0 | 0 | 0 | 0 | 0 | 0 | No evidence of MC, collagenous colitis, or IBD |
| 13 | 2/2002 | 6/2019 | ***6/2013**  **Fecal incontinence** | **0** | **1** | **1** | **1** | **1** | **0** | **1** | **Colonic mucosa with acute cryptitis, glandular atrophy and architectural distortion in background of organizing fibrosis. Findings are suggestive of a long standing/chronic, possibly resolving injury. IBD appears less likely given lack of significant lymphoplasmacytic infiltrate.** |
|  |  |  | *12/2021  UC history | 1 | 0 | 0 | 0 | n/a (only polyps biopsied) | n/a | n/a |  |

* denotes biopsy specimens that were available for direct evaluation by our gastrointestinal pathologist

Bold = on PPS, not bold = off PPS

CD = Crohn’s disease

E = eosinophils

IBD = inflammatory bowel disease

MC = microscopic colitis

N = neutrophils

UC = ulcerative colitis

**Supplemental Table 2.** Exposures to other IC treatments and additional potential risk factors according to presence or absence of IBD, and IBD or IBS (cross-sectional study).

| **Covariate** | **IBD** | | | **IBD or IBS** | | | |
| --- | --- | --- | --- | --- | --- | --- | --- |
|  | **Yes**  **(n=19)**  **Mean ± SD or n (%)** | **No**  **(n=200)**  **Mean ± SD or n (%)** | **Crude^1^ (C) and Adjusted^2^ (A) Odds Ratios (95% CI), P-value** | **Yes**  **(n=34)**  **Mean ± SD or n (%)** | | **No**  **(n=185)**  **Mean ± SD or n (%)** | **Crude^1^ (C) and Adjusted^2^ (A) Odds Ratios (95% CI), P-value** |
| Hydroxyzine | | | | | | | |
| Yes (n=56) | 7 (13%) | 49 (87%) | C: 1.8 (0.6, 5.3), 0.27 | 13 (23%) | 43 (77%) | | C: 2.0 (0.9, 4.4), 0.07 |
| No (n=163) | 12 (7%) | 151 (93%) |  | 21 (13%) | 142 (87%) | |  |
| Tricyclic antidepressants | | | | | | | |
| Yes (n=64) | 5 (8%) | 59 (92%) | C: 0.9 (0.2, 2.5), 0.77 | 13 (20%) | 51 (80%) | | C: 1.6 (0.8, 3.5), 0.21 |
| No (n=155) | 14 (9%) | 141 (91%) |  | 21 (14%) | 134 (86%) | |  |
| Gabapentin or pregabalin | | | | | | | |
| Yes (n=88) | 9 (10%) | 79 (90%) | C: 1.4 (0.5, 3.5), 0.50 | 15 (17%) | 73 (83%) | | C: 1.2 (0.6, 2.5), 0.61 |
| No (n=131) | 10 (8%) | 121 (92%) |  | 19 (15%) | 112 (85%) | |  |
| Hydroxychloroquine | | | | | | | |
| Yes (n=13) | 0 (0%) | 13 (100%) | C: 0.0 (0.0, 2.8), 0.61 | 2 (15%) | 11 (85%) | | C: 1.0 (0.1, 4.9), 1.00 |
| No (n=206) | 19 (9%) | 187 (91%) |  | 32 (16%) | 174 (84%) | |  |
| Cyclobenzaprine | | | | | | | |
| Yes (n=55) | 3 (5%) | 52 (95%) | C: 0.5 (0.1, 2.0), 0.42 | 8 (15%) | 47 (85%) | | C: 0.9 (0.4, 2.1), 0.82 |
| No (n=164) | 16 (10%) | 148 (90%) |  | 26 (16%) | 138 (84%) | |  |
| Methenamine | | | | | | | |
| Yes (n=31) | 1 (3%) | 30 (97%) | C: 0.3 (0.01, 2.2), 0.49 | 3 (10%) | 28 (90%) | | C: 0.5 (0.1, 1.9), 0.43 |
| No (n=188) | 18 (10%) | 170 (90%) |  | 31 (16%) | 157 (84%) | |  |
| Phenazopyridine | | | | | | | |
| Yes (n=53) | 2 (4%) | 51 (96%) | C: 0.3 (0.03, 1.5), 0.17 | 4 (8%) | 49 (92%) | | C: 0.4 (0.1, 1.1), 0.07 |
| No (n=166) | 17 (10%) | 149 (90%) |  | 30 (18%) | 136 (82%) | |  |
| Oxybutynin | | | | | | | |
| Yes (n=30) | 1 (3%) | 29 (97%) | C: 0.3 (0.01, 2.2), 0.48 | 2 (7%) | 28 (93%) | | C: 0.3 (0.01, 2.2), 0.48 |
| No (n=189) | 18 (10%) | 171 (90%) |  | 32 (17%) | 157 (83%) | |  |
| Isotretinoin | | | | | | | |
| Yes (n=1) | 0 (0%) | 1 (100%) | C: 0.0 (0.0, 200.0), 1.0 | 1 (100%) | 0 (0%) | | C: ∞ (0.29, ∞), 0.16 |
| No (n=218) | 19 (9%) | 199 (91%) |  | 33 (15%) | 185 (85%) | |  |
| Oral contraceptives | | | | | | | |
| Yes (n=52) | 6 (12%) | 46 (88%) | C: 1.5 (0.5, 4.7), 0.40 | 9 (17%) | 43 (83%) | | C: 1.2 (0.5, 2.7), 0.68 |
| No (n=167) | 13 (8%) | 154 (92%) |  | 25 (15%) | 142 (85%) | |  |
| Mycophenolate mofetil | | | | | | | |
| Yes (n=4) | 0 (0%) | 4 (100%) | C: 0.0 (0.0, 12.1), 1.00 | 0 (0%) | 4 (100%) | | C: 0.0 (0.0, 6.1), 1.00 |
| No (n=215) | 19 (9%) | 196 (91%) |  | 34 (16%) | 181 (84%) | |  |
| Etanercept | | | | | | | |
| Yes (n=0) | 0 | 0 |  | 0 | 0 | |  |
| No (n=219) | 19 (9%) | 200 (91%) |  | 34 (16%) | 185 (84%) | |  |
| Ipilimumab | | | | | | | |
| Yes (n=0) | 0 | 0 |  | 0 | 0 | |  |
| No (n=219) | 19 (9%) | 200 (91%) |  | 34 (16%) | 185 (84%) | |  |
| Rituximab | | | | | | | |
| Yes (n=1) | 0 (0%) | 1 (100%) | C: 0.0 (0.0, 200.0), 1.0 | 0 (0%) | 1 (100%) | | C: 0.0 (0.0, 103.4), 1.00 |
| No (n=218) | 19 (9%) | 199 (91%) |  | 34 (16%) | 184 (84%) | |  |
