# Supplementary material for: Colopathy associated with pentosan polysulfate use
Source: Front Pharmacol. 2025 Jul 28;16:1494467. doi: 10.3389/fphar.2025.1494467 (PMC12336275; doi:10.3389/fphar.2025.1494467)
Supplement: Supplementary file 2 [file Image1.pdf]

Study Overview: Two-Part Investigation into PPS Associated Colopathy

Part One: Cohort Study

- Well-characterized cohort of 13 long-term PPS users
- 11/13 (85%) developed IBD symptoms after using PPS
    - 9 diagnosed with IBD, 2 with IBS
- 10/10 with slides reviewed had histologic abnormalities
- **2 required colectomy for dysplasia**
- Overall improvement after PPS cessation

Part Two: Cross-Sectional Study

- 219 patients with interstitial cystitis
    - 80 with PPS exposure (36.5%)
- PPS exposure significant predictor of IBD diagnosis [adjusted odds ratio = 3.3 ($p = 0.02$)]
- No other medication associated with the IBD outcome

Summary

- Strong association between PPS use and IBD
- Suggests novel drug-induced colopathy, requiring colectomy in some cases
- Further research needed to assess causality

Supplemental Figure 1. Overview of study design and results.
PPS = pentosan polysulfate; IBD = inflammatory bowel disease; IBS = irritable bowel syndrome
